# BCG‐Related Immune Reconstitution Inflammatory Syndrome and Delayed Donor Chimerism After Allogeneic HSCT in T^−^B^−^NK^+^ SCID: A Successful Report of Steroid‐Sparing Management

**DOI:** 10.1155/crii/3971651

**Published:** 2026-07-27

**Authors:** Seyed Reza Abdipour Mehrian, Sarah Ravankhah, Kiarash Kavari, Elahe Meftah, Hadis Jafarian, Somayeh Khajeh, Hadi Mottaghipisheh, Ali Amanati

**Affiliations:** ^1^ Clinical Research Development Center, Amir Oncology Teaching Hospital, Shiraz University of Medical Sciences, Shiraz, Iran, sums.ac.ir; ^2^ Yasuj University of Medical Sciences, Yasuj, Iran, yums.ac.ir; ^3^ Student Research Committee, Kerman University of Medical Sciences, Kerman, Iran, kmu.ac.ir; ^4^ Professor Alborzi Clinical Microbiology Research Center, Shiraz University of Medical Sciences, Shiraz, Iran, sums.ac.ir; ^5^ Hematology Research Center, Shiraz University of Medical Sciences, Shiraz, Iran, sums.ac.ir

**Keywords:** Bacillus Calmette–Guérin (BCG) disease, hematopoietic stem cell transplantation, immune reconstitution inflammatory syndrome, mixed chimerism, severe combined immunodeficiency

## Abstract

**Background:**

Severe combined immunodeficiency (SCID) is a life‐threatening disorder that requires early hematopoietic stem cell transplantation (HSCT). In regions where Bacillus Calmette–Guérin (BCG) vaccination is routinely administered at birth, disseminated BCG disease significantly complicates the clinical course in infants. Post‐HSCT, immune reconstitution inflammatory syndrome (IRIS) poses an additional challenge, particularly in patients with active mycobacterial infections and mixed chimerism.

**Case Presentation:**

We describe a female infant with T^−^B^−^NK^+^ SCID who developed disseminated BCG disease after routine BCG vaccination. At 6 months, she underwent HSCT from an HLA‐matched sibling without a conditioning regimen, according to the EBMT/ESID guidelines for T^−^B^−^NK SCID. Donor chimerism was undetectable on two early occasions (days +14 and +28), initially raising concerns of graft failure. During this window, the patient developed paradoxical BCG‐related IRIS, progressive lymphadenitis, and fever, which ultimately coincided with the first emergence of donor cells rather than graft loss. Corticosteroids were withheld because of the risk of infection and rejection. Instead, escalation of multidrug antimycobacterial therapy (intensified by adding amikacin and clarithromycin) led to clinical improvement. Subsequent testing demonstrated stable mixed donor chimerism (21% at +40, 23% at +60, and 51% at 8 months) with progressive donor T‐cell reconstitution and the absence of graft‐versus‐host disease (GVHD). At the most recent follow‐up, ~16 months after HSCT, the patient remained clinically well.

**Conclusion:**

This case highlights the diagnostic and therapeutic dilemmas in managing SCID patients with BCG disease after HSCT. Early negative chimerism does not always predict graft failure, and paradoxical IRIS should be considered when clinical deterioration coincides with immune recovery after HSCT. Our findings suggest that conservative corticosteroid‐sparing management with intensified antimycobacterial therapy may improve outcomes.

## 1. Introduction

Severe combined immunodeficiency (SCID) is a congenital disorder characterized by defective T‐cell development, often accompanied by B‐cell and natural killer (NK) cell deficits. Newborns with SCID may appear healthy but develop severe infections within a few months of birth. Without intervention, SCID is fatal; thus, hematopoietic stem cell transplantation (HSCT) is the main curative option [1–3]. Early HSCT, preferably before 3.5 months of age and prior to major infection, markedly improves survival [1, 4]. In regions where Bacillus Calmette–Guérin (BCG) vaccination is routine, such as the WHO Eastern Mediterranean Region, undiagnosed SCID can lead to disseminated BCG infections, complicating HSCT and increasing mortality. Studies have shown that, if not properly treated, BCG disease can significantly increase transplant‐related mortality [5, 6]. During immune recovery, immune reconstitution inflammatory syndrome (IRIS) may worsen latent infections [7]. Although immune reconstitution indicates successful engraftment, it may also mask or aggravate pre‐existing infections, such as BCG. Thus, careful posttransplant surveillance is crucial. BCG‐related IRIS in patients undergoing immunological recovery following engraftment can potentially damage the host and graft depending on treatment failure, resistance, or superposed infection [8, 9]. Another complexity in post‐HSCT management is mixed chimerism, which may affect the pace and quality of immune reconstitution and influence infection control and the risk of IRIS.

This report provides an in‐depth account of an infant with SCID and BCG disease who underwent HSCT and subsequently developed BCG‐related IRIS. The following case highlights the diagnostic and therapeutic challenges associated with BCG‐related IRIS, particularly in the context of mixed chimerism.

## 2. Case Presentation

The patient, a female infant born at 36 weeks, was the second live birth to consanguineous first‐cousin parents. There was no family history of primary immunodeficiency.

For clarity, the day of HSCT is defined as day 0 in the following description, and subsequent time points are expressed as day +X after HSCT (and, when relevant, as negative days before HSCT).

At 3 months of age, corresponding to approximately day −90 relative to HSCT, she developed left axillary lymphadenopathy at the BCG injection site, which progressively enlarged and eventually ulcerated.

The lesion was positive for acid‐fast bacilli and the *Mycobacterium tuberculosis* complex by PCR. Species‐ and strain‐level confirmation was obtained by region‐of‐difference (RD) PCR: deletion of RD9 placed the organism within the *M. bovis* branch of the complex, excluding *M. tuberculosis*, and deletion of RD1—retained by virulent *M. bovis*—identified the isolate specifically as *M. bovis* BCG. Mycobacterial culture and phenotypic drug susceptibility testing were not available at our center; therefore, identification relied on smear microscopy and molecular confirmation (MTB‐complex PCR and RD1/RD9 deletion analysis), and partial drug resistance could not be excluded using phenotypic methods [10, 11].

Antimycobacterial therapy was initiated for the patient. Soon after discharge, she developed respiratory distress and required ICU admission but without improvement. The patient was referred to Namazi Hospital (Shiraz) for advanced treatment. On admission, she exhibited failure to thrive (a weight of 4.4 kg at 3 months of age), fever, hepatosplenomegaly, and poor oral intake. Immunological workup revealed severe T‐ and B‐cell lymphopenia with preserved NK cells (CD3^+^: 2.77%, CD4^+^: 2.35%, CD8^+^: 0.43%, CD16/56^+^: 78.4%, and CD19^+^: 0.72%). Baseline laboratory tests revealed preserved organ function, and ultrasonography confirmed lymphadenitis (Table 1). Normal neutrophil oxidative burst (DHR), normal CD11b expression, and preserved IFN‐γ receptor expression excluded chronic granulomatous disease, leukocyte adhesion deficiency, and a defect in the IFN‐γ/IL‐12 axis (Mendelian susceptibility to mycobacterial disease), respectively, supporting SCID as the basis for susceptibility to disseminated BCG infection.

**Table 1:** Baseline laboratory findings at presentation, including lymphocyte immunophenotyping (confirming a T^−^B^−^NK^+^ SCID phenotype), neutrophil function, hematology, biochemistry, and inflammatory markers.

Parameter	Result	Reference range (age‐appropriate)	Interpretation
Lymphocyte immunophenotyping (initial diagnostic sample; % of lymphocytes, absolute count in parentheses)			
CD3^+^ (total T cells)	2.77% (37/µL)	51%–77%	Markedly decreased
CD3^+^CD4^+^ (helper T cells)	2.35% (32/µL)	35%–56%	Markedly decreased
CD3^+^CD8^+^ (cytotoxic T cells)	0.43% (6/µL)	12%–23%	Severely decreased
CD19^+^ (B cells)	0.72% (10/µL)	11%–41%	Severely reduced
CD16^+^/CD56^+^ (NK cells)	78.4% (1058/µL)	3%–14%	Relative NK predominance (preserved)
CD3^-^CD16^+^CD56^+^ (true NK cells)	67.84% (915/µL)	—	Preserved
CD4:CD8 ratio	5.46	1.2–3.5	Elevated (secondary to low CD8)
Neutrophil/innate immune function			
DHR (neutrophil oxidative burst)	194	>50 (normal); 10–50 borderline; <10 abnormal	Normal oxidative burst; excludes chronic granulomatous disease
CD11b expression (neutrophils)	99%	Normal	Normal; argues against leukocyte adhesion deficiency
IFN‐γ receptor expression	67%	Present	Present; argues against an IFN‐γ/IL‐12 axis defect (MSMD)
Hematology			
White blood cell count	13,490/µL	4,000–11,000/µL	Borderline/mildly elevated (infant ranges are higher)
Biochemistry			
Blood urea nitrogen	3 mg/dL	5–20 mg/dL	Mildly low
Creatinine	0.22 mg/dL	0.2–0.7 mg/dL	Normal
Sodium	136 mEq/L	135–145 mEq/L	Normal
Potassium	6.3 mEq/L	3.5–5.5 mEq/L	Elevated—most consistent with a hemolyzed specimen (pseudohyperkalemia); clinical correlation recommended
Calcium (total)	9.5 mg/dL	8.8–10.8 mg/dL	Normal
Glucose (serum)	60 mg/dL	70–100 mg/dL	Mildly low
Total protein	5.9 g/dL	5.7–8.0 g/dL	Low‐normal
Albumin	3.9 g/dL	3.5–5.0 g/dL	Normal
Globulin	2.0 g/dL	2.0–3.5 g/dL	Low‐normal (consistent with humoral immunodeficiency)
AST	48 U/L	≤45 U/L	Mildly elevated
ALT	48 U/L	≤31 U/L	Mildly elevated (mild transaminitis)
Total bilirubin	0.19 mg/dL	0.3–1.3 mg/dL	Low/normal
Direct bilirubin	0.04 mg/dL	≤0.3 mg/dL	Normal
Alkaline phosphatase	498 U/L	180–1200 U/L	Normal (physiologically high in infancy)
Creatine phosphokinase	35 U/L	30–200 U/L	Normal
LDH	990 U/L	≤850 U/L	Elevated
Uric acid	3.1 mg/dL	2.0–5.5 mg/dL	Normal
Inflammatory markers			
C‐reactive protein	2.0 mg/L	≤6 mg/L	Not elevated

*Note:* Lymphocyte subsets are expressed as percentages of lymphocytes (absolute counts in parentheses). Age‐appropriate lymphocyte‐subset reference ranges are per Shearer et al. [12] and the performing laboratory; hematology and biochemistry intervals are age‐matched laboratory ranges or standard pediatric intervals. The T^−^B^−^NK^+^ immunophenotype is diagnostic of SCID; normal neutrophil oxidative burst (DHR), normal CD11b expression, and preserved IFN‐γ receptor expression exclude chronic granulomatous disease, leukocyte adhesion deficiency, and an IFN‐γ/IL‐12 axis defect (MSMD), respectively.

Abbreviations: DHR, dihydrorhodamine; IFN, interferon; IL, interleukin; LDH, lactate dehydrogenase; MSMD, Mendelian susceptibility to mycobacterial disease; NK, natural killer; SCID, severe combined immunodeficiency.

Genetic testing identified a homozygous DCLRE1C deletion consistent with the Athabascan‐type SCID.

By the time of transplant at 6 months of age, she had received antimycobacterial therapy for approximately 3 months (since the diagnosis at 3 months of age). At that point, her respiratory status had improved, with no further ICU‐level support, she was afebrile, and her oral intake and weight had stabilized, although BCG lymphadenitis persisted. The patient was considered clinically stable enough to proceed with HSCT.

At 6 months of age (day 0), the patient underwent unconditioned HSCT from a healthy 14‐year‐old HLA‐identical (10/10) sibling donor by high‐resolution typing (HLA‐A, ‐B, ‐C, ‐DRB1, and ‐DQB1). Conditioning was omitted because T^−^B^−^NK^+^ SCID with an HLA‐identical sibling donor permits engraftment without it, in accordance with EBMT guidance; graft‐versus‐host disease (GVHD) prophylaxis was withheld given the low GVHD risk in this setting, to avoid blunting donor engraftment in the context of anticipated mixed chimerism, and because additional immunosuppression could have exacerbated the active BCG infection [13]. The graft was an unmanipulated peripheral blood stem cell product (no T‐cell depletion or CD34 selection), with a total volume of 17 mL containing 8.66 × 10^6^ CD34^+^ cells and 215 × 10^6^ CD3^+^ cells (mononuclear cell fraction 70%), corresponding to ~2.0 × 10^6^ CD34^+^ cells/kg and 49 × 10^6^ CD3^+^ cells/kg at a body weight of 4.4 kg.

The recipient had blood group B Rh‐negative, and the donor had blood group B Rh‐positive (a minor ABO‐compatible, Rh‐mismatched pairing). The recipient was cytomegalovirus (CMV)‐seropositive before transplantation (CMV IgG reactive, IgM negative), as was the donor (CMV IgG‐positive), representing a seropositive recipient with a seropositive donor (R+/D+).

Notably, GVHD did not develop despite an unmanipulated graft with a substantial CD3^+^ T‐cell dose and no prophylaxis.

Following HSCT, the patient remained febrile and clinically unstable during the early posttransplant period. Supportive care included immunoglobulin replacement, *Pneumocystis jirovecii* pneumonia (PCP) and antifungal prophylaxis, CMV/Epstein–Barr virus (EBV) surveillance, and irradiated, leukoreduced blood products.

Donor chimerism was quantified by multiplex quantitative fluorescence PCR (QF‐PCR) using 17 polymorphic short tandem repeat (STR) markers. Amplicons were separated on an ABI 3100 Genetic Analyzer and analyzed with GeneMarker software by comparing donor and recipient STR profiles. All reported values represent total (whole‐blood) donor chimerism in unsorted peripheral blood; lineage‐specific (T‐cell, B‐cell, NK‐cell, or myeloid) chimerism was not assessed.

Because the transplant was unconditioned, there was no neutropenic period and hence no classic donor neutrophil engraftment milestone; peripheral blood neutrophils were present throughout and were therefore predominantly of host origin, consistent with the early whole‐blood donor chimerism remaining below the detection threshold. In this setting, donor engraftment is expected to be T‐lineage‐restricted, which whole‐blood chimerism may underestimate.

**Figure 1:**
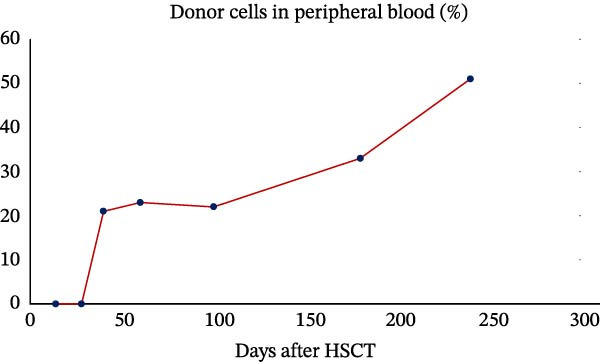
Trend of donor chimerism (% donor cells in peripheral blood) over time from day +14 to 8 months after HSCT, measured by multiplex QF‐PCR using polymorphic STR markers. Donor chimerism was undetectable (<1%, below the assay detection limit) on days +14 and +28, rose to 21% on day +40 shortly after the clinical flare consistent with BCG‐related IRIS, was 23% on day +60, 22% on day +100, 33% on day +180, and reached 51% by 8 months (day +240). HSCT, hematopoietic stem cell transplantation; QF‐PCR, quantitative fluorescence polymerase chain reaction; STR, short tandem repeat.

Genomic DNA was extracted from peripheral blood in the same laboratory using the same method at every timepoint. The assay’s lower limit of detection for a minor population was ~1%; therefore, values reported as 0% denoted donor chimerism below this threshold rather than the confirmed absence of donor cells [14]. On days +14 and +28, donor chimerism was undetectable (<1%), raising concerns regarding graft failure. Around day +18, she developed a marked inflammatory flare characterized by painful swelling and drainage at the BCG lymphadenitis site. Shortly thereafter, repeat chimerism on day +40 showed the first emergence of mixed donor chimerism at ~21% of donor cells. This pattern, together with localized worsening at a known BCG focus under ongoing antimycobacterial therapy, led to a working diagnosis of BCG‐induced IRIS. Antimycobacterial therapy was intensified, and donor chimerism subsequently remained in the mixed range (23% on day +60, 22% on day +100, and 33% on day +180) and rose to 51% by 8 months post‐HSCT (day +240), the last formal chimerism measurement, with progressive donor T‐cell reconstitution with persistent B‐cell deficiency requiring ongoing immunoglobulin replacement, and no evidence of GVHD (Figure 1).

BCG‐related IRIS was supported by a previously documented disseminated BCG infection under ongoing multidrug antimycobacterial therapy, acute inflammatory worsening localized to the previous BCG lesion, and a temporal association with emerging donor immune reconstitution after HSCT. At this time, early chimerism studies had shown undetectable donor cells (below the assay threshold), but shortly after the flare, donor chimerism became detectable and subsequently increased, which was more consistent with immune recovery than graft failure.

Around the inflammatory flare and the early posttransplant period (approximately days +18 to +40), alternative explanations for the deterioration were systematically evaluated (Table 2). All infectious investigations were negative, no features of GVHD developed at any point, and the only site of clinical progression was the known BCG focus. Together with the temporal coincidence between this localized worsening and the first emergence of donor chimerism, this pattern supports paradoxical BCG‐related IRIS rather than an alternative infection, GVHD, or graft failure.

**Table 2:** Systematic evaluation of alternative explanations for the posttransplant inflammatory deterioration, with the investigations performed and their outcomes.

Alternative diagnosis	Investigation(s) performed	Result/conclusion
Bacterial sepsis	Blood cultures; inflammatory markers (CRP, ESR)	Cultures negative; inflammatory markers inconsistent with bacterial sepsis—excluded
Fungal infection	Fungal cultures and antigen testing; earlier tissue evaluation	Negative—excluded
Viral reactivation	Cytomegalovirus and Epstein–Barr virus surveillance	Negative—excluded
Drug reaction/hypersensitivity	Clinical review of temporal relationship to medications; eosinophil count	No temporal association with a new drug; no eosinophilia—considered unlikely
Graft‐versus‐host disease (GVHD)	Clinical assessment (skin, gastrointestinal, hepatic) throughout follow‐up	No clinical or laboratory features of GVHD at any time—did not develop
Progressive/disseminated BCG disease	Clinical course; site and pattern of progression; response under therapy; chimerism trajectory	Worsening localized to the known BCG focus (not disseminating) and coinciding with first‐emerging donor chimerism—considered less likely than paradoxical IRIS

*Note:* Investigations were performed around the inflammatory flare and the early posttransplant period (approximately days +18 to +40 relative to HSCT); exact dates of individual tests could not be reliably reconstructed from the available records and are therefore given approximately. All infectious investigations were negative and graft‐versus‐host disease did not develop at any point; the only site of clinical progression was the known BCG focus. Together with the temporal coincidence between this localized worsening and the first emergence of donor chimerism, this pattern supported paradoxical BCG‐related immune reconstitution inflammatory syndrome (IRIS) rather than an alternative infection, GVHD, or graft failure.

Abbreviations: BCG, bacille Calmette–Guérin; CRP, C‐reactive protein; ESR, erythrocyte sedimentation rate; GVHD, graft‐versus‐host disease; HSCT, hematopoietic stem cell transplantation; IRIS, immune reconstitution inflammatory syndrome.

The primary antimycobacterial regimen comprised isoniazid 10 mg/kg/day (50 mg once daily), rifampicin 10 mg/kg/day (50 mg once daily), ethambutol 20 mg/kg/day (100 mg once daily), and linezolid 10 mg every 8 h (50 mg thrice daily). At the time of the inflammatory flare, therapy was intensified by the addition of clarithromycin (7.5 mg/kg every 12 h [35 mg twice daily]) and amikacin (15 mg/kg/day [65 mg once daily]). Supportive care included immunoglobulin replacement (from 1 week before HSCT, weekly until 2 months posttransplant, then every 4 weeks), PCP and antifungal prophylaxis, CMV and EBV surveillance, and the use of irradiated and leukoreduced blood products.

Within a week, donor chimerism increased to 23%, and clinical recovery commenced. Corticosteroids were avoided to reduce the risks of infection and rejection. The total transplant admission length of stay was 47 days (day −7 to day +40), and the patient was discharged once donor chimerism became detectable and the inflammatory flare had settled. Antimycobacterial therapy was continued beyond discharge with isoniazid, rifampicin, and ethambutol.

During follow‐up, she showed steady improvement with weight gain (7 kg, 70 cm, 4 months after HSCT) and normal development. Donor chimerism continued to rise, reaching 51% by 8 months, with the gradual resolution of lymphadenopathy. Aside from a self‐limited lower respiratory tract infection and oral candidiasis, there was no recurrence of BCG or other serious infections.

At the most recent assessment, ~16 months after HSCT (at 22 months of age), she remained clinically well, with sustained donor T‐cell reconstitution, continued absence of GVHD, and ongoing immunoglobulin replacement therapy for persistent B‐cell deficiency.

Serial lymphocyte subset phenotyping from before HSCT through ~16 months post‐HSCT showed a progressively expanding CD4‐predominant T‐cell compartment with a corresponding decline in the initially dominant NK cell fraction, consistent with donor T‐cell reconstitution; the B‐cell (CD19^+^) compartment remained markedly low throughout (Table 3).

**Table 3:** Serial peripheral‐blood lymphocyte‐subset phenotyping by flow cytometry, from before HSCT through ~16 months posttransplant.

Day^a^	Age (months)	CD3^+^ (% lymph)	CD4^+^/CD8^+^ (% of T)	CD4:CD8	CD19^+^ (% lymph)	NK CD16/CD56 (% lymph)
−7 (baseline)	6	14	66/34	1.9	0.3	76/62
+90	9	40	70/25	3.5	0.26	36/32
+165	11	17.4	80.5/13.5	6.0	1.25	26.5/10
+228	13	34.8	77.3/16.5	4.7	1.1	18.5/12.7
+347	17	72.4	77.2/17.7	4.36	1.3	13.8/9.7
+480	22	71	72/20	3.6	1.0	22/17

Abbreviations: HSCT, hematopoietic stem cell transplantation; NK, natural killer.

^a^Timing is expressed as days relative to HSCT (day 0) to preserve anonymity; the day −7 sample is the pretransplant baseline. CD3^+^, CD19^+^, CD16^+^ and CD56^+^ are percentages of lymphocytes; CD4^+^ and CD8^+^ are percentages of T cells. NK‐cell markers CD16 and CD56 are reported separately; they identify overlapping NK‐cell populations and are not additive. Where a same‐day complete blood count was available (days −7 and +165; absolute lymphocyte count 1100/µL), approximate absolute counts were: day −7—CD3 154, CD4 102, CD8 52, CD19 3, NK ≈680–840/µL; day +165—CD3 191, CD4 154, CD8 26, CD19 14/µL. Immunoglobulins at day +165: IgG 1431 mg/dL (on immunoglobulin replacement), IgM 21 mg/dL (low), IgA 32 mg/dL (low), IgE 1,823 IU/mL (high).

Baseline immunoglobulin levels were markedly reduced (IgG 33, IgM 3, and IgA 4 mg/dL), and the patient remained on immunoglobulin replacement (intravenous immunoglobulin from 1 week before HSCT, weekly until 2 months posttransplant, then every 4 weeks), consistent with the persistently low B‐cell compartment.

## 3. Discussion

In this case report, we describe an infant with T^−^B^−^NK^+^ SCID who developed paradoxical BCG‐related IRIS after allogeneic HSCT, highlighting the diagnostic and therapeutic challenges associated with mycobacterial complications during immune reconstitution. Because no specific biomarker exists for BCG‐related IRIS, we interpreted the clinical course using the INSHI consensus framework for paradoxical mycobacterial IRIS adapted to the post‐HSCT setting [15, 16], which requires clinical or radiological worsening of a treated mycobacterial infection after immune recovery in the absence of a plausible alternative diagnosis. In our patient, the key supportive features were proven disseminated BCG disease already under multidrug antimycobacterial therapy, acute exacerbation of fever and lymphadenitis at the previous BCG site shortly after HSCT coinciding with the first emergence of donor‐derived chimerism, and a comprehensive negative workup for fungal, bacterial, and other opportunistic infections. Conversely, primary graft failure, GVHD, drug hypersensitivity, and a new unrelated infection were unlikely as donor chimerism subsequently increased, no clinical signs of GVHD appeared, and the child improved while continuing and intensifying antimycobacterial therapy.

Another possibility was a persistent or partially drug‐resistant BCG infection rather than IRIS. Because mycobacterial culture and phenotypic drug susceptibility testing were unavailable at our center, this possibility could not be excluded microbiologically and was instead weighed on clinical grounds. The inflammatory worsening was localized to the prior BCG focus rather than disseminating, and it coincided closely with the first emergence of donor chimerism, features more consistent with paradoxical immune recovery than with uncontrolled progressive infection. Continued clinical improvement, including after intensification of antimycobacterial therapy, was supportive but, on its own, not discriminating since improvement after escalation could also follow the control of a previously failing regimen.

Taken together, these elements strongly support a diagnosis of paradoxical BCG‐related IRIS triggered by early donor immune recovery, although persistent infection cannot be excluded from the differential diagnosis. To contextualize this paradoxical inflammatory reaction, it is important to consider the underlying immunodeficiency and its standard treatments. SCID is a life‐threatening disorder caused by profound T‐cell dysfunction, often with absent or defective B and NK cells [1, 2]. HSCT remains the only curative therapy, with the best outcomes achieved when it is performed before severe infections [4]. In countries with newborn screening for T‐cell receptor excision circles (TRECs), early diagnosis enables timely transplantation [3, 17, 18]. In contrast, in regions where newborn screening is not available, delayed recognition often leads to life‐threatening complications, including disseminated BCG disease after routine vaccination [17, 19]. Such infections increase the risk of transplant‐related morbidity and create substantial diagnostic and therapeutic dilemmas during the post‐HSCT period.

The major diagnostic challenge in this case was differentiating between primary graft failure, progressive infection, and paradoxical IRIS. Two early chimerism tests showing undetectable donor cells (below the ~1% detection limit of the STR‐based QF‐PCR assay), together with concurrent fever and lymphadenitis, initially suggested graft failure, consistent with previously published reports [18]. However, the EBMT Handbook emphasizes that early negative chimerism, especially in unconditioned SCID transplants, should not be considered definitive evidence of graft failure since engraftment may occur later, may be lineage‐specific, and may not immediately correlate with clinical recovery [20].

In unconditioned matched‐sibling SCID transplants, donor T‐cell engraftment can be achieved without conditioning; however, because there is no myeloablation, there is typically no neutropenic nadir or conventional neutrophil engraftment milestone, and myeloid and B‐lineage engraftment are characteristically low or absent. Donor T‐cell reconstitution develops over months rather than days, commonly with measurable circulating donor T cells within the first 3–6 months and progressive recovery over the first 1–2 years. Durable thymopoiesis (reflected in TREC counts by ~6 months) predicts robust long‐term reconstitution. Persistent B‐cell deficiency requiring long‐term immunoglobulin replacement is common in this setting [21–23].

In our case, donor lymphocyte infusion (DLI) was withheld because of concerns regarding clinical instability and the risk of severe GVHD. Subsequent donor engraftment of 23% highlights the danger of premature conclusions based solely on the early results of chimerism.

In patients with active BCG disease and suspected IRIS, the cornerstone of management is prolonged multidrug antimycobacterial therapy, whereas systemic corticosteroids or other immunomodulators are generally reserved for severe or life‐threatening inflammatory complications because of concerns regarding uncontrolled infection and graft compromise. Our steroid‐sparing approach aligns with recent case reports and series of BCG‐related IRIS after HSCT in SCID and other primary immunodeficiencies, which consistently emphasize antimycobacterial therapy as the primary intervention and report highly heterogeneous, case‐based use of systemic steroids and other anti‐inflammatory agents [13, 18, 24–29].

A complete diagnostic workup was performed to exclude fungal superinfection, bacterial sepsis, and other opportunistic pathogens, all of which were negative results. This systematic approach was essential because both IRIS and true progressive infection can present with overlapping clinical features, including fever and lymphadenitis [30]. Only after the exclusion of alternative diagnoses could paradoxical IRIS be reliably considered. Indeed, paradoxical IRIS is increasingly recognized in patients with SCID and BCG disease following HSCT, typically presenting with inflammatory exacerbation at BCG sites during early donor immune recovery [30]. Our patient’s simultaneous clinical deterioration and the delayed emergence of donor chimerism provide a textbook example of this phenomenon.

The interpretation of chimerism trends remains unresolved. While the EBMT guidelines recommend monitoring at fixed time points (days +14, +28, +60, +100, and +180), there is no consensus regarding the optimal frequency of repeat testing once negative results are obtained. Published case series have highlighted significant variability, reflecting the absence of a standardized approach [5, 19, 20, 31].

This gap leaves clinicians to rely heavily on integrated clinical and laboratory judgments. Our case study highlights the significance of considering chimerism as a dynamic trajectory that should be interpreted in connection with infection workup and clinical progression rather than as a static binary marker of successful versus failed engraftment. Making therapeutic decisions in such situations remains difficult. Although corticosteroids are often advised in paradoxical IRIS to reduce inflammation [19], their use is debatable in cases of active mycobacterial infection because immunosuppression may make it more difficult to eliminate the bacteria [32]. The patient responded to intensification of antimycobacterial therapy with the addition of amikacin and clarithromycin to the primary regimen of isoniazid, rifampicin, ethambutol, and linezolid rather than corticosteroids or DLI. This is consistent with previous case studies and Latin American consensus guidelines that emphasize long‐term multidrug treatment as the mainstay for managing BCG disease in patients with impaired immune systems [5, 17, 32]. Taken together, our experience suggests three practical points for clinicians caring for SCID patients with BCG disease after HSCT. First, early negative chimerism in unconditioned SCID transplants should not automatically be interpreted as graft failure; donor engraftment may occur later and should be interpreted as a trajectory in conjunction with clinical findings. Second, new or worsening BCG site inflammation during immune recovery should trigger a structured differential that distinguishes progressive infection from paradoxical IRIS and graft failure based on repeat microbiological workups, imaging, and serial chimerism monitoring rather than chimerism alone. Third, in patients with active BCG disease, a conservative, steroid‐sparing strategy that prioritizes prolonged, intensified multidrug antimycobacterial therapy and close follow‐up may be a reasonable first‐line approach, reserving systemic corticosteroids or other immunomodulators for life‐threatening inflammatory complications after excluding alternative diagnoses.

**Figure 2:**
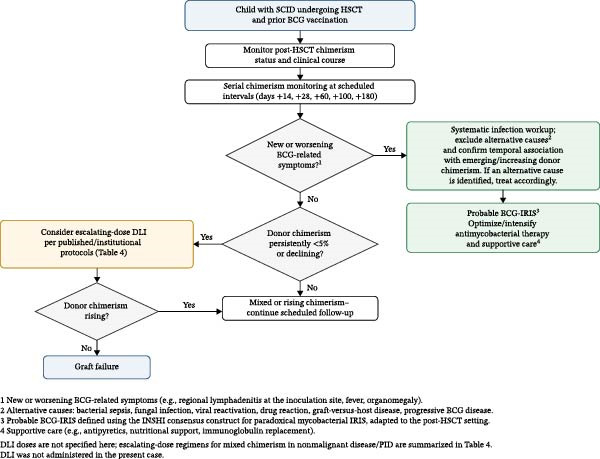
Proposed framework for monitoring and managing donor chimerism and BCG‐related complications in a child with SCID undergoing HSCT after prior BCG vaccination. Donor chimerism is monitored at scheduled intervals (days +14, +28, +60, +100, and +180). New or worsening BCG‐related symptoms prompt a systematic infection workup with exclusion of alternative causes and confirmation of a temporal association with emerging or increasing donor chimerism; when the pattern fits, probable BCG‐IRIS (defined using the INSHI consensus construct for paradoxical mycobacterial IRIS, adapted to the post‐HSCT setting) is managed by optimizing or intensifying antimycobacterial therapy and supportive care. Persistently low (<5%) or declining donor chimerism prompts consideration of escalating‐dose donor lymphocyte infusion (DLI) per published or institutional protocols (Table 4); failure to respond indicates graft failure. DLI was not administered in the present case. Although developed for SCID, this framework may inform the management of disseminated BCG disease in other inborn errors of immunity undergoing HSCT. BCG, Bacillus Calmette–Guérin; DLI, donor lymphocyte infusion; HSCT, hematopoietic stem cell transplantation; IRIS, immune reconstitution inflammatory syndrome; SCID, severe combined immunodeficiency.

These principles are operationalized in the diagnostic and therapeutic frameworks, as shown in Figure 2. The algorithm begins with a SCID patient with known or suspected BCG disease who deteriorates after HSCT and first mandates the exclusion of alternative infections, GVHD, and drug toxicity. In the presence of documented progressive BCG disease or a new pathogen, the framework directs clinicians to escalate antimycobacterial or pathogen‐specific therapies. When no alternative cause is identified, donor chimerism dynamics guide the next step: persistently absent or declining chimerism supports graft failure and consideration of DLI or a second HSCT, whereas localized worsening at previous BCG sites coinciding with emerging or increasing donor chimerism supports a diagnosis of BCG‐related IRIS. In this scenario, maintaining or intensifying combination antimycobacterial therapy while avoiding routine high‐dose corticosteroids may permit both infection control and donor immune recovery, although this approach remains based on case‐level evidence and should be individualized.

**Table 4:** Published escalating‐dose donor lymphocyte infusion (DLI) regimens for mixed chimerism in nonmalignant disease and primary immunodeficiency.

Setting (reference)	Indication	Starting CD3^+^ dose (cells/kg)	Escalation scheme	Maximum/cumulative dose	Interval and stopping rule
Pediatric nonmalignant disease (Gabelli et al. [33])	Mixed chimerism in nonmalignant disease	1 × 10^7^	1 × 10^7^ → 5 × 10^7^ → 1 × 10^8^ (max. 3 doses)	Up to 1 × 10^8^	Fixed minimum interval; stop on GVHD or attainment of full donor chimerism
PID after reduced‐intensity conditioning (Tahani et al., [34])	Mixed chimerism in primary immunodeficiency	Escalating (stepwise)	Stepwise (≈half‐log) increments as tolerated	Max. ≈ 3.6 × 10^7^; cumulative ≈ 9.3 × 10^7^ (median 3.2 doses)	Begun at median day +105; GVHD the main dose‐limiting toxicity
General principles (pooled experience)	Mixed/declining donor chimerism	Low starting dose	Escalate only if the prior dose is tolerated without GVHD	Individualized	Withhold/stop on GVHD or full donor chimerism; lineage‐specific chimerism may guide timing

*Note:* Doses are expressed as CD3^+^ cells/kg of recipient body weight. DLI was not administered in the present case; this table summarizes published regimens to support the escalating‐DLI step shown in Figure 2. The optimal starting dose, escalation, and interval are not standardized and should follow institutional protocols, with graft‐versus‐host disease the principal dose‐limiting toxicity. Regimens adapted from references [33] and [34].

Abbreviations: GVHD, graft‐versus‐host disease; PID, primary immunodeficiency.

Decisions about DLI are deliberately restricted to children with persistently low or declining donor chimerism and controlled infection; in that setting, escalating‐dose DLI has been used to convert mixed to full donor chimerism in nonmalignant diseases and primary immunodeficiency, with GVHD as the principal dose‐limiting toxicity. Reported regimens are summarized in Table 4 [33, 34].

This study had several limitations. First, as a single case, the findings cannot be generalized to all SCID patients with BCG disease, and our proposed algorithm remains a hypothesis‐generating tool for future studies.

Second, the diagnosis of BCG‐related IRIS relied on clinical judgment and consensus criteria rather than a specific confirmatory biomarker, and although serial lymphocyte subset phenotyping was available, we lacked naïve/memory T‐cell characterization and T‐cell functional assays that might have further clarified the underlying immunopathology.

Third, neither mycobacterial culture nor phenotypic drug susceptibility testing was available at our center; although RD‐PCR confirmed the organism as *M. bovis* BCG, partial drug resistance could not be excluded by microbiological means (weighed in the differential reasoning earlier).

Fourth, donor chimerism was monitored by QF‐PCR on peripheral blood (without lineage separation), with a detection limit for a minor population of ~1% and without lineage‐specific analysis; a more sensitive platform (quantitative or digital droplet PCR, ~0.01%–0.1%) and/or lineage‐specific chimerism, particularly CD3^+^ T‐cell chimerism, the most informative compartment in unconditioned T^−^, might have detected low‐level or T‐lineage‐restricted donor engraftment earlier, before it became measurable by whole‐blood chimerism.

Consequently, our steroid‐sparing, multidrug antimycobacterial strategy should be interpreted cautiously and individualized according to local expertise and resources.

## 4. Conclusion

This case illustrates that early undetectable donor chimerism (below the QF‐PCR detection limit) in unconditioned SCID HSCT does not necessarily indicate graft failure and that paradoxical BCG‐related IRIS should be considered when clinical deterioration coincides with the first emergence of donor chimerism. Our experience suggests that a conservative corticosteroid‐sparing strategy with intensified multidrug antimycobacterial therapy, combined with careful interpretation of serial chimerism, may be a reasonable option in similar settings, as illustrated by the sustained clinical recovery and donor T‐cell reconstitution maintained for ~16 months in our patient.

### 4.1. Patient Perspective

The patient’s parents described the experience as emotionally distressing, particularly after the unexpected diagnosis of SCID and the complications following routine BCG vaccination. Persistent fever, worsening lymphadenitis, and early reports of 0% donor chimerism caused the family considerable anxiety regarding the possibility of graft failure and their child’s prognosis. They appreciated the clear communication and shared decision‐making regarding the diagnosis of IRIS and the decision to intensify antimycobacterial therapy while avoiding corticosteroid use.

Gradual clinical improvement, weight gain, and the absence of new infections reassured the family and restored their confidence in the treatment. They expressed hope that sharing their child’s experience would increase awareness of early SCID diagnosis and improve the care of similar patients.

**Nomenclature**
SCID: Severe combined immunodeficiency
HSCT: Hematopoietic stem cell transplantation
BCG: Bacillus Calmette–Guérin
IRIS: Immune reconstitution inflammatory syndrome
NK: Natural killer
AFB: Acid‐fast bacilli
PCR: Polymerase chain reaction
QF‐PCR: Quantitative fluorescence polymerase chain reaction
STR: Short tandem repeat
RD: Region of difference
DHR: Dihydrorhodamine
CGD: Chronic granulomatous disease
MSMD: Mendelian susceptibility to mycobacterial disease
IFN: Interferon
IL: Interleukin
LDH: Lactate dehydrogenase
CMV: Cytomegalovirus
EBV: Epstein–Barr virus
PCP: *Pneumocystis jirovecii* pneumonia
IVIG: Intravenous immunoglobulin
GVHD: Graft‐versus‐host disease
DLI: Donor lymphocyte infusion
EBMT: European Society for Blood and Marrow Transplantation
ESID: European Society for Immunodeficiencies
TRECs: T‐cell receptor excision circles
ICU: Intensive care unit
INSHI: International Network for the Study of HIV‐associated IRIS.

## Author Contributions

Seyed Reza Abdipour Mehrian collected clinical data and drafted the first version of the manuscript. Sarah Ravankhah contributed to the literature review and drafting and revising the manuscript. Kiarash Kavari contributed to the acquisition and interpretation of clinical and laboratory data and assisted in data collection. Elahe Meftah collected clinical data and critically revised the manuscript. Hadis Jafarian and Somayeh Khajeh supervised the microbiological investigations and interpreted the related data. Hadi Mottaghipisheh supervised hematologic and transplant management, interpreted hematology/transplant‐related data, and critically revised the manuscript for important intellectual content. Ali Amanati designed the study, coordinated clinical management, supervised infectious disease care, and critically revised the manuscript for important intellectual content.

## Funding

No funding was received for this manuscript.

## Disclosure

All authors have read and approved the final manuscript.

## Ethics Statement

This case report was conducted in accordance with the national regulations governing medical research in Iran and the principles of medical ethics. Ethical approval was granted by the Institutional Review Board (IRB) of the Shiraz University of Medical Sciences (Approval ID: IR.SUMS.REC. 1404.519) [35].

## Consent

The patient received standard clinical care, and no experimental interventions were performed. Written informed consent for the publication of clinical details and images was obtained from the patient’s parents/legal guardians. Informed consent was obtained from the patient and/or the patient’s legal guardians after a full explanation of the study.

## Conflicts of Interest

The authors declare no conflicts of interest.

## Data Availability

The data that support the findings of this study are available from the corresponding author upon reasonable request.
